# Levels and Seasonal Trends of C_1_–C_4_ Perfluoroalkyl Acids and the Discovery of Trifluoromethane Sulfonic Acid in Surface Snow in the Arctic

**DOI:** 10.1021/acs.est.1c04776

**Published:** 2021-11-15

**Authors:** Maria K. Björnsdotter, William F. Hartz, Roland Kallenborn, Ingrid Ericson Jogsten, Jack D. Humby, Anna Kärrman, Leo W. Y. Yeung

**Affiliations:** † Man-Technology-Environment Research Centre (MTM), Örebro University, Örebro SE-701 82, Sweden; ‡ Department of Earth Sciences, University of Oxford, South Parks Road, Oxford OX1 3AN, United Kingdom; § Department of Arctic Geology, University Centre in Svalbard (UNIS), Longyearbyen, Svalbard NO-9171, Norway; ∥ Faculty of Chemistry, Biotechnology and Food Sciences (KBM), Norwegian University of Life Sciences (NMBU), Ås NO-1432, Norway; ⊥ Department of Arctic Technology, University Centre in Svalbard (UNIS), Longyearbyen, Svalbard NO-9171, Norway; # Ice Dynamics and Paleoclimate, British Antarctic Survey, High Cross, Cambridge CB3 0ET, United Kingdom

**Keywords:** perfluoroalkyl substances, ultrashort-chain perfluoroalkyl acids, trifluoroacetic acid, solar radiation, atmospheric oxidation, atmospheric deposition, precursors, Arctic

## Abstract

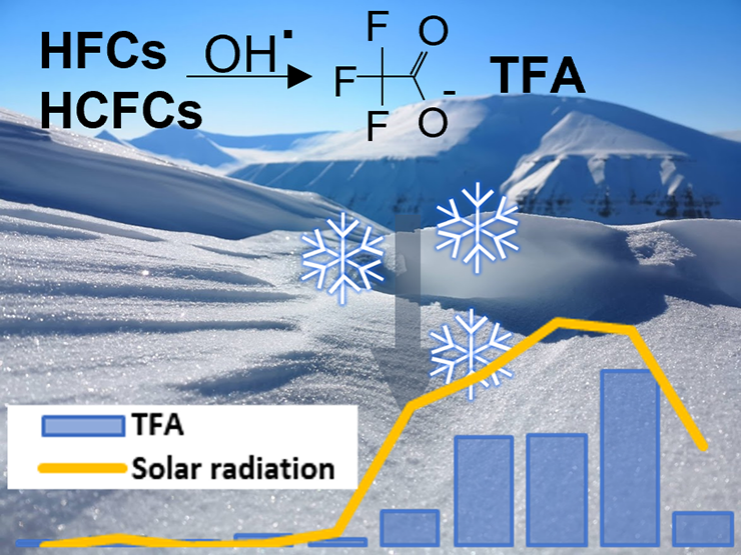

C_1_–C_4_ perfluoroalkyl acids (PFAAs) are highly persistent chemicals that have been found in the environment. To date, much uncertainty still exists about their sources and fate. The importance of the atmospheric degradation of volatile precursors to C_1_–C_4_ PFAAs were investigated by studying their distribution and seasonal variation in remote Arctic locations. C_1_–C_4_ PFAAs were measured in surface snow on the island of Spitsbergen in the Norwegian Arctic during January–August 2019. Trifluoroacetic acid (TFA), perfluoropropanoic acid (PFPrA), perfluorobutanoic acid (PFBA), and trifluoromethane sulfonic acid (TFMS) were detected in most samples, including samples collected at locations presumably receiving PFAA input solely from long-range processes. The flux of TFA, PFPrA, PFBA, and TFMS per precipitation event was in the ranges of 22–1800, 0.79–16, 0.19–170, and 1.5–57 ng/m^2^, respectively. A positive correlation between the flux of TFA, PFPrA, and PFBA with downward short-wave solar radiation was observed. No correlation was observed between the flux of TFMS and solar radiation. These findings suggest that atmospheric transport of volatile precursors and their subsequent degradation plays a major role in the global distribution of C_2_–C_4_ perfluoroalkyl carboxylic acids and their consequential deposition in Arctic environments. The discovery of TFMS in surface snow at these remote Arctic locations suggests that TFMS is globally distributed. However, the transport mechanism to the Arctic environment remains unknown.

## Abstract 2

Atmospheric oxidation of volatile precursors is a major source of C_2_−C_4_ perfluoroalkyl carboxylic acids in Arctic environments.

## Introduction

Perfluoroalkyl acids (PFAAs) are highly persistent, man-made chemicals that are ubiquitous in the environment. C_1_–C_4_ PFAAs are perfluoroalkyl carboxylic acids (PFCAs) and perfluoroalkyl sulfonic acids (PFSAs) with alkyl chain lengths of one to four carbon atoms. These include trifluoroacetic acid (TFA), perfluoropropanoic acid (PFPrA), perfluorobutanoic acid (PFBA), trifluoromethane sulfonic acid (TFMS), perfluoroethane sulfonic acid (PFEtS), perfluoropropane sulfonic acid (PFPrS), and perfluorobutane sulfonic acid (PFBS). The acidic functional group, in combination with the small molecular structure, results in highly polar compounds. High concentrations in biological matrices have not been reported, but due to the persistence to degradation, precautionary measures should be taken.^1^ TFA can accumulate in aquatic environments,^2^ and contamination of drinking water has been shown.^3^

TFA is a transformation product of hydrofluorocarbons (HFCs) and hydrochlorofluorocarbons (HCFCs),^4^ which were introduced as replacements of the ozone-depleting chlorofluorocarbons used as cooling agents after the introduction of the Montreal protocol in 1989. TFA has been studied since the early 1990s and has been frequently reported in the environment since then. TFA seems to be ubiquitous in surface snow even at very remote sites,^5,6^ and its environmental concentrations are increasing.^7^ It has been frequently reported in the scientific literature that the environmental levels of TFA from the breakdown of HFCs, HCFCs, and hydrofluoro-olefins (HFOs), which are recently introduced replacements due to their lower global warming potential, do not pose a threat to humans or the environment.^8−11^ TFA is a substance of multiple sources that are still uncertain, and it is a potential degradation product of more than one million chemicals.^10^ Continued attention is needed considering the long environmental lifetime.

Among the C_1_–C_4_ PFCAs, PFPrA and PFBA have not been studied as thoroughly as TFA, yet they have also been reported in surface snow and surface water.^12−15^ TFA, PFPrA, and PFBA were recently reported in ice caps in remote locations^16^ showing that they are globally distributed, including across polar regions. The formation of C_1_–C_4_ PFAAs from the atmospheric degradation of volatile precursors is today considered as a pathway to remote locations. Such precursors include fluorotelomer alcohols, perfluoroalkane sulfonamides, and perfluoroalkane sulfonamidoethanols, of which organofluorine compounds with C_4_ to C_14_ chain lengths have been frequently detected in the Arctic.^17−21^ In addition, thermolysis of fluoropolymers used in consumer products result in direct and indirect formation of PFCAs.^22^ While direct formation mainly led to elevated concentrations locally, the indirect formation can result in global distribution because of the long-range atmospheric transport of volatile intermediates. Another transport mechanism to remote locations is via marine aerosols, which involves the transfer of PFAAs into the atmosphere from the ocean via sea spray formation as a result of strong wind and breaking waves.^23^

The formation of TFA, PFPrA, and PFBA from the degradation of precursors has been demonstrated under laboratory conditions.^24−26^ These degradations were initiated by the light-dependent formation of hydroxyl radicals. Through a hydroxyl radical-mediated unzipping cycle, longer-chain >C_4_ precursor compounds can contribute to the atmospheric formation of C_1_–C_4_ PFAAs.^24^ Modeling studies found Arctic atmospheric PFOA concentrations, as a result of 8:2 fluorotelomer alcohol atmospheric degradation, to be 15–20 times higher during July compared to January.^27^ This was attributed to the large seasonal variations in radiation causing a seasonal variation in the atmospheric hydroxyl radical concentration. Other potential atmospheric precursor sources are HFCs and HCFCs. These have been detected globally, including in the Arctic.^28^ PFAAs have a short atmospheric half-life of several days with respect to wet deposition and are effectively scavenged by wet deposition.^29,30^ Hence. during snowfall, it would be expected that almost all atmospheric PFAAs become deposited, regardless of the type of source. Sampling fresh snowfall can therefore offer a route to understanding atmospheric PFAA processes.

One study has investigated the formation of PFBS from the degradation of *N*-methyl perfluorobutane sulfonamidoethanol,^25^ but data about the formation of C_1_–C_4_ PFSAs from volatile precursors is still scarce and little is known about their global distribution. A few studies have reported C_1_–C_4_ PFSAs in wastewater^31^ and in surface water and groundwater that were connected to suspected point sources such as landfills, military training sites, and waste management facilities.^15,32^ A recent study reported TFMS in surface water and groundwater far away from primary environmental emission points.^33^ The potential sources and the environmental fate of these substances are not yet well understood. However, with respect to their high polarity and high persistence, contamination in the aqueous environment could be expected.

Local sources of PFAAs in the Arctic have also been identified. On Spitsbergen in the Norwegian Arctic, PFBA and PFBS have been linked with local sources in the settlement of Longyearbyen such as a firefighting training site (FFTS) and a landfill.^34,35^ PFBS was found in one snow sample (and at several trophic levels in local biota). In another study, PFBA was detected in the snow in Longyearbyen.^36^ Otherwise, there has been no further study into the sources and processes of C_1_–C_4_ PFAAs in snow in the Arctic.

The aim of the present study was to assess the seasonal deposition, sources, and geographical distribution of C_1_–C_4_ PFAAs in the Arctic. For this purpose, seven PFAAs, namely TFA, PFPrA, PFBA, TFMS, PFEtS, PFPrS, and PFBS, were measured in surface snow samples collected at several locations on the island of Spitsbergen in the Norwegian Arctic, including around the settlement of Longyearbyen. The correlation between the observed flux and solar radiation was examined to elucidate the relevance of the atmospheric degradation of volatile precursors as a pathway to remote locations.

## Materials and Methods

### Chemicals and Reagents

Native standards of PFBA, PFPrS, and PFBS and mass-labeled standards of PFBA and PFBS were purchased from Wellington Laboratories (Guelph, ON, Canada). TFA was purchased from Sigma-Aldrich (Munich, Germany). PFPrA was from Sigma-Aldrich (Oakville, ON, Canada). TFMS was from Sigma-Aldrich (Stockholm, Sweden), and potassium salt of PFEtS was obtained from Kanto Chemical Co., Inc. (Portland, OR, USA). Mass-labeled standard of TFA (^13^C_2_-TFA) was purchased from Toronto Research Chemicals Inc. (Toronto, ON, Canada). Mass-labeled standards for PFPrA, TFMS, PFEtS, and PFPrS were not available commercially. The purity of all standards was above 97%. Ammonium acetate (≥99.0%) was purchased from Sigma-Aldrich (Stockholm, Sweden). Glacial acetic acid (EMPROVE EXPERT, Ph. Eur., JP, USP) was purchased from Merck (Darmstadt, Germany). Analytical reagent-grade ammonia solution, HPLC grade methanol (≥99.8%), and LC–MS-grade methanol (≥99.9%) were from Fisher Scientific (Ottawa, ON, Canada).

### Sample Collection

**Figure 1:**
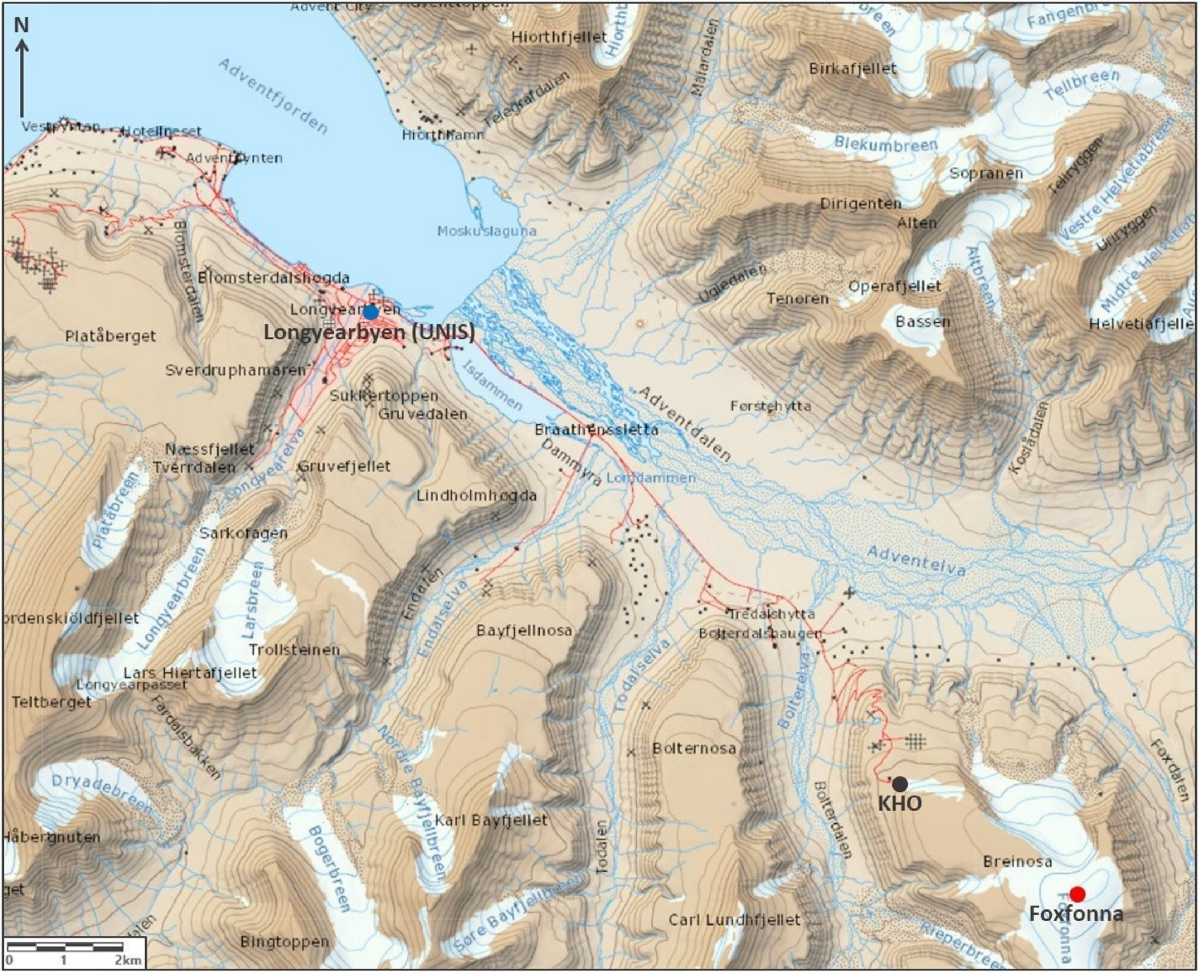
Sampling locations in Longyearbyen (*n* = 8), Kjell Henriksen Observatory (KHO, *n* = 9), and Foxfonna (*n* = 10). The map was reproduced from TopoSvalbard, Norwegian Polar Institute.

Seven sampling locations were chosen for surface snow sampling. Three sites were sampled several times from January 2019 to August 2019. These three sampling sites were chosen to represent a range of potential locally contaminated and background sites while being easily accessible all year round to allow for seasonal sampling. These three sites were in the settlement of Longyearbyen (*n* = 8; 78°13.288′N 15°39.041′E; 13 m above sea level), up the hill from the Kjell Henriksen Observatory (KHO; *n* = 9; 78°08.807′N 16°02.781′E; 532 m above sea level), and on the summit of the Foxfonna ice cap (*n* = 10; 78°07.736′N 16°10.791′E; 800 m above sea level). The sampling locations are shown in Figure 1.

The site in Longyearbyen was located in a fenced-off area of tundra outside the University Centre in Svalbard (UNIS) which, while being in the center of town, was a location where the surface snow remained undisturbed by traffic or pedestrians. This site was 360 m from the fjord’s coastline. It was chosen to investigate local sources and its possible associated seasonal variations. The site at KHO was located 150 m away uphill of the Kjell Henriksen Observatory. This site was chosen due to its proximity to the Foxfonna sampling site (4.7 km away), allowing for direct comparison between the sites and to understand possible PFAA contamination from the active coal mine in Longyearbyen (1.3 km from the KHO sampling site). The Foxfonna sampling site was chosen to represent a potential remote location due to its high altitude (800 m above sea level) and position upwind from Longyearbyen (16 km) with respect to the easterly prevailing winds. Sampling at the site in Longyearbyen was conducted eight times from January 2019 to May 2019 (samples UNIS01–UNIS08). Sampling at KHO was conducted nine times from January 2019 to June 2019 (KHO01–KHO09). Sampling at the Foxfonna site was conducted 10 times from January 2019 to August 2019 (samples Fox01–Fox10). Sampling at these three sites was conducted as soon as possible after a chosen precipitation event (typically <1–7 days for the Foxfonna sampling site, average 3.7 days), such that each snow sample represents a single precipitation event where postdepositional processes have been minimized.

**Figure 2:**
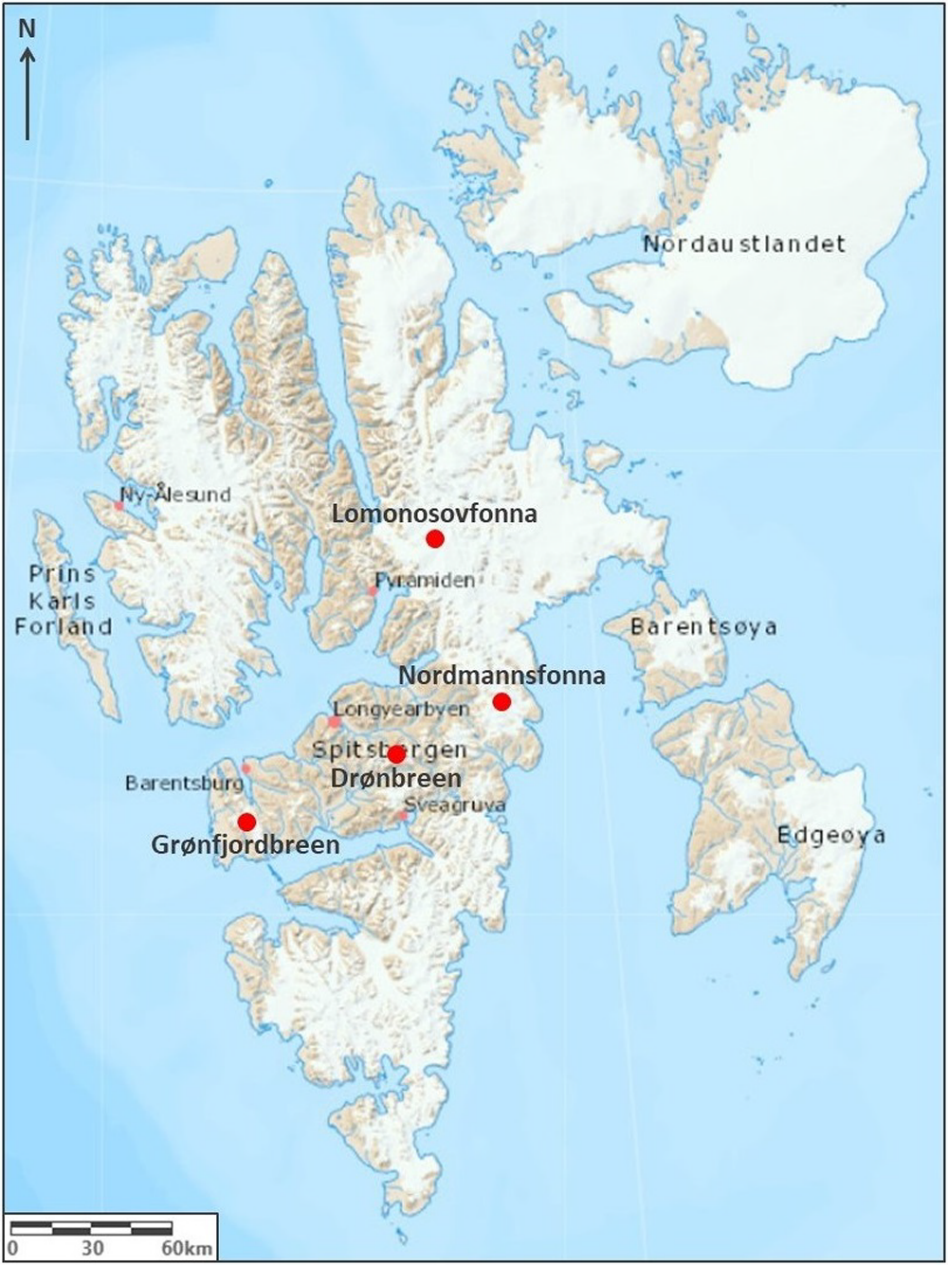
The reference snow sampling locations on Spitsbergen glaciers: Lomonosovfonna (*n* = 2), Nordmannsfonna (*n* = 1), Drønbreen (*n* = 1), and Grønfjordbreen (*n* = 1). The map was reproduced from TopoSvalbard, Norwegian Polar Institute.

Four high elevation sites on glaciers around Spitsbergen were chosen for snow sampling as these most likely represented background reference locations presumably receiving PFAA input solely from atmospheric long range processes (Figure 2). Sampling was conducted at these sites February to April 2019 at Drønbreen (*n* = 1; 78°06.185′N 16°39.182′E; 707 m above sea level), Lomonosovfonna (*n* = 2; 78°49.454′N 17°26.253′E; 1198 m above sea level), Grønfjordbreen (*n* = 1; 77°53.222′N 14°13.745′E; 574 m above sea level) and Nordmannsfonna (*n* = 1; 78°15.894′N 18°23.717′E; 498 m above sea level).

When collecting the surface snow samples, the site was approached by ski or snowmobile, with the final approach to the sampling site undertaken from downwind on foot. Nitrile gloves were worn during snow sampling. PFAAs have been shown to be effectively scavenged from the atmosphere by wet deposition, and most of the PFAA removal is expected to occur at the beginning of rainfall.^30^ A similar process during a snow precipitation event is likely, and the initial snowfall would presumably have higher PFAA concentrations. However, due to windy conditions in Svalbard, it was assumed that the snow was well mixed by both snow blowing in the atmosphere and surface snow drifting before it became deposited, and hence, the PFAA concentrations within the fresh snowfall were homogeneous. A precleaned aluminum shovel was used to collect the upper 0–5 cm of the fresh surface snow into a precleaned high-density polyethylene barrel. The sampling area for the 32 surface snow samples was between 0.8–3.9 m^2^ (average 1.8 m^2^). The barrel was then sealed and transported back to UNIS, where it was melted at 5 °C, bottled into precleaned polypropylene containers, and stored in a refrigerator at 2–4 °C. The samples were then transported cold to Örebro University, Sweden and stored at 8 °C until further processing.

### Sample Preparation and Analysis

All samples were ultrasonicated for 10 min to desorb target analytes possibly sorbed to the inner surface of the containers. The inner surface of the containers was rinsed with methanol once the sample was taken out for filtration, and the methanol was combined with the sample. Surface snow samples were filtered with glass microfiber filters (Whatman, Grade GF/B, 1.2 μm) prior to extraction. The filters were extracted three times with methanol by ultrasonication for 30 min followed by centrifugation at 8000 rpm for 5 min. The filter extract was combined with the water sample. The pH of the samples was adjusted to 4 by addition of acetic acid prior to extraction.

Approximately 2200 mL of sample was extracted by weak anion exchange solid-phase extraction (Oasis WAX, Waters Corporation, Milford, USA) following the ISO25101 method with some modifications. The cartridges were preconditioned with 4 mL of 0.1% ammonium hydroxide in methanol followed by 4 mL of methanol and 4 mL of Milli-Q water. The samples were then loaded at approximately 1 drop/s onto the cartridges. After loading the samples, the cartridges were washed by passage of 4 mL of Milli-Q water followed by 4 mL of ammonium acetate buffer solution (pH 4) and then dried under vacuum for 30 min before elution of target analytes. The neutral fraction was eluted by adding 4 mL of methanol. The anionic fraction was then eluted by adding 4 mL of 0.1% ammonium hydroxide in methanol. In the present study, only the anionic fraction was analyzed. The eluate was evaporated to approximately 0.5 mL at 60 °C and 400 mbar, transferred to an LC vial, and then further evaporated to 100 μL under a gentle stream of nitrogen. Mass-labeled internal standards (1 ng) were added to the samples prior to filtration to monitor the recovery of the method. Aliquots of 2 μL were injected into the supercritical fluid chromatography (SFC) tandem mass spectrometry system (MS/MS) for quantification of C_1_–C_4_ PFAAs.

Separation and quantification were performed using SFC-MS/MS (Acquity Ultra Performance Convergence Chromatograph and Xevo TQ-S micro, Waters Corporation, Milford, MA, USA) operated in negative electrospray ionization mode. An SFC Torus DIOL column (3.0 mm diameter, 150 mm length, 1.7 μm particle size; Waters Corporation, Milford, MA, USA) maintained at 50 °C was used to achieve chromatographic separation. The mobile phase consisted of CO_2_ (A) and 0.1% ammonium hydroxide in methanol (B). The gradient of the mobile phase started with an initial B concentration of 15%, which was then increased to 35% over 5 min. This was held for 1 min before returning to initial conditions over 1 min. The flow rate was 1.2 mL/min, and the active back pressure regulator was set at 2000 psi throughout the chromatographic separation. The source parameters were set as follows: capillary voltage, 0.7 kV; source temperature, 150 °C; desolvation temperature, 400 °C; cone gas flow, 150 L/h; desolvation gas flow, 800 L/h; collision gas flow, 0.2 mL/min; nebulizer, 6.5 bar. Two transitions were monitored for TFMS, PFEtS, PFPrS, and PFBS, respectively. Only one transition was monitored for TFA, PFPrA, and PFBA. MRM transitions for all target analytes are provided in Table S1.

### Statistical Analysis

Spearman rank correlations between the PFAA flux in surface snow and solar radiation as well as between the TFMS flux and the flux of sodium ions were calculated using Microsoft Excel version 2105.

### Quality Assurance and Quality Control

Linear regression analysis showed good linearity for each analyte (*R*^2^ > 0.99) in the range from 2 to 100 ng/mL. Instrumental limits of quantification (LOQ) were set as the lowest calibration point with a signal-to-noise ratio of at least 10. The repeatability of the analytical method was evaluated based on repeated injections (*n* = 10) of a standard with a concentration of 4 ng/mL. The relative standard deviation of repeated injections was in the range of 0.33 to 4.1%. Isotope dilution was used for quantification. For those target analytes that did not have corresponding mass-labeled standards, the homolog closest in retention time was used for quantification (Table S1). Extraction efficiencies were assessed based on the peak area of native standards spiked to test samples (*n* = 3) after subtraction of the background concentrations in the samples. For TFA, the extraction efficiency was assessed based on the peak area of a mass-labeled standard spiked to test samples (*n* = 3). The extraction efficiency was in the range of 58–126%. The repeatability of the extraction method was evaluated based on the relative standard deviation of the spiked test samples (*n* = 3) at a concentration of 1 ng per 250 mL sample. The relative standard deviation of the spiked test samples was in the range of 2.2–15%. The method limits of quantification (MQLs) were calculated as the average concentration in repeated blank extractions (*n* = 5) plus three times the standard deviation. For those analytes that were not observed in blank extractions, the instrument LOQ was used as the MDL. A field blank was included to ensure that no contamination occurred during sampling. The field blank comprised purified water in a sample container that was brought to the field and opened during the time of the sampling. The container was resealed and transported to the laboratory, where it was treated in the same way as the samples. None of the target analytes were observed in the field blank (TFA, <0.009 ng/L; PFPrA, <0.009 ng/L; PFBA, <0.058 ng/L; TFMS, <0.009 ng/L; PFEtS, <0.05 ng/L; PFPrS, <0.009 ng/L; PFBS, <0.01 ng/L). Detailed information about the precision of the analytical method, extraction efficiencies and repeatability, and LOQ and MQL are provided in Table S2.

### Air Mass Trajectories and Solar Radiation

At the Foxfonna sampling site, the Hybrid Single-Particle Lagrangian Integrated Trajectory (HYSPLIT) model was used to assess whether downward short-wave radiation may be linked to precursor degradation and, hence, the flux of C_1_–C_4_ PFAAs. The National Center for Environmental Prediction’s Global Data Assimilation System (GDAS) model was used for the meteorological input data. A single backward 6 day air mass trajectory was calculated for each of the 10 snow sampling rounds, which ended at the Foxfonna sampling site (at 800 m above sea level). The trajectory was timed to end at the onset of the precipitation event that was subsequently sampled. The onset of the precipitation event was established by inspecting meteorological data from the Adventdalen weather station (11.4 km from the Foxfonna sampling site; data was retrieved from the Norwegian Meteorological Institute). An integrated radiation value was then calculated from each trajectory by summing the downward short-wave radiation associated with each hourly point on the trajectory. This served as a record of the downward short-wave radiation each air mass parcel had been subject to that was associated with each snow sample (in kWh/m^2^). The atmospheric lifetime of C_1_–C_4_ PFAAs are assumed to be short, and its removal is dominated by wet- and dry-deposition similar to other strong acids such as nitric acid.^29^ A 6 day backward air mass trajectory was used, which is the global average half-life of nitric acid with respect to wet and dry deposition.

## Results and Discussion

### C_1_–C_4_ PFAAs in Surface Snow

Detailed information about the PFAA concentrations measured in surface snow is provided in Tables S3–S6, and the deposition fluxes of PFAAs per precipitation event are provided in Tables S7–S10. TFA, PFPrA, and TFMS were detected in all surface snow samples, including those collected at the reference locations. The flux was in the ranges of 22–1800, 0.79–16, and 1.5–57 ng/m^2^ for TFA, PFPrA, and TFMS, respectively. PFBA was detected in 97% of the surface snow samples and in all samples collected at reference locations at fluxes in the range of 0.19–170 ng/m^2^. PFPrS was not detected in any of the samples. Neither PFEtS nor PFBS were detected in samples collected at the remote sites. PFEtS was not detected at the Foxfonna sampling site but was detected in 22% of the samples collected at KHO and in all samples collected in Longyearbyen (UNIS) at fluxes in the range of 0.52–55 ng/m^2^. PFBS was not detected at locations outside of the Longyearbyen settlement (Foxfonna and KHO) but was detected in 63% of the samples collected in Longyearbyen in the concentration range of 0.17–26 ng/m^2^. The detection of PFEtS and PFBS in surface snow samples in Longyearbyen is likely due to local sources. PFBS has previously been reported in runoff from a FFTS and in landfill leachate in Longyearbyen,^34,35^ and PFEtS has been linked to similar sources.^15,32^ The median flux of PFBA was eight times higher in Longyearbyen (UNIS) (34 ng/m^2^) compared to Foxfonna (4.1 ng/m^2^). This indicates that local sources of PFBA exist within the settlement.

TFA, PFPrA, PFBA, and TFMS were all detected at Foxfonna sampling site and at the four high-elevation reference sites (Drønbreen, Lomonosovfonna, Grønfjordbreen, and Nordmannsfonna), which are thought to represent input solely from long-range processes. The observed flux was highest for TFA ranging from 22 to 1800 ng/m^2^ at the Foxfonna sampling site. PFPrA, PFBA, and TFMS were detected at the Foxfonna sampling site at fluxes ranging from 0.79–16 ng/m^2^ (PFPrA), <0.99–20 ng/m^2^ (PFBA), and 2.2–11 ng/m^2^ (TFMS). The concentrations of TFA (5.6–270 ng/L), PFPrA (0.21–1.5 ng/L), and PFBA (0.10–10 ng/L) in surface snow is in the same range as previously reported in precipitation in urban areas,^13,31,37−44^ and similar concentrations of TFA has previously been reported in precipitation in remote locations.^6^ A significant correlation was observed between the flux of TFA and PFPrA (*r* = 0.93, *P* < 0.01), TFA and PFBA (*r* = 0.85, *P* < 0.01), and PFPrA and PFBA (*r* = 0.81, *P* < 0.01) in samples collected at the Foxfonna sampling site (Table S11). These findings suggest that TFA, PFPrA, and PFBA share similar atmospheric sources and fate in remote Arctic environments.

It has been suggested that marine aerosols play an important role in the global distribution of TFA.^45^ No significant correlation was observed between TFA, PFPrA, and PFBA with sodium (Table S11). Previous work with remote snow samples have also observed this lack of correlation for C_2_–C_4_ PFCAs.^46^ The lack of correlations observed in the present study supports the observation by Pickard et al. (2018) that marine aerosol inputs are unimportant to the long-range transport of C_2_–C_4_ PFCAs to remote Arctic environments.

### Seasonal Variation of TFA, PFPrA, and PFBA in Surface Snow

To investigate seasonal variations in long range processes, data from the Foxfonna ice cap was used since this offered the longest time series. As the fluxes of TFA, PFPrA, and PFBA from the snow samples at the reference sites were of a similar range as the fluxes in the snow samples from Foxfonna, equivalent long-range processes are dominating the explanation for the presence of these compounds in the snow samples from Foxfonna. This evidence, combined with Foxfonna’s high elevation site upwind of Longyearbyen with respect to the prevailing winds, means that this site offers the best potential for a remote site of the three main sampling locations.

The observed median fluxes of TFA, PFPrA, and PFBA in the surface snow on Foxfonna were higher by 36, 6, and 2 times for TFA, PFPrA and PFBA, respectively, during precipitation event sampling in April–August 2019 (rounds 6–10, samples Fox06–Fox10) compared to sampling in January–March 2019 (rounds 1–5, samples Fox01–Fox05). This sampling was done during the polar summer period with 24 h daylight in April–August (Fox06–Fox10) and compared with samples collected during the polar night with complete (24 h) or partial (0–10 h daylight) darkness in January–March (Fox01–Fox05). These results are in line with previously modeled results, which found that the atmospheric concentrations of PFOA, having formed via hydroxyl radical mediated degradation of 8:2 FTOH, would be 15–20 times higher in the Arctic in July compared to January.^27^ This was attributed to seasonal variations in atmospheric hydroxyl radical concentrations. The amplitude of the seasonal variations for the different PFAAs observed might be linked to different sources.

To inspect this relationship further, HYSPLIT modeling was used to simulate the relationship between atmospheric hydroxyl radical concentrations and the flux of precursor degradation products (in this instance TFA, PFPrA, and PFBA in surface snow). Since hydroxyl radicals are produced by incoming solar radiation into the atmosphere,^47^ the downward short-wave radiation was used to access the seasonal variations in hydroxyl radicals in the Arctic atmosphere. A backward air mass trajectory of 6 days from the time of sampling was used to represent the air mass that brought the precipitation event that was consequently sampled in the surface snow at Foxfonna (Figure S1). A backward air mass trajectory with a length of 6 days was used, since it was expected that that atmospheric half-life of TFA with respect to wet/dry deposition would be similar to that of nitric acid.^29^ Therefore, the majority of TFA observed in the surface snow would be expected to come from precursor degradations that occurred during the 6 day transport of this air mass parcel that carried the precipitation event to Foxfonna.

**Figure 3:**
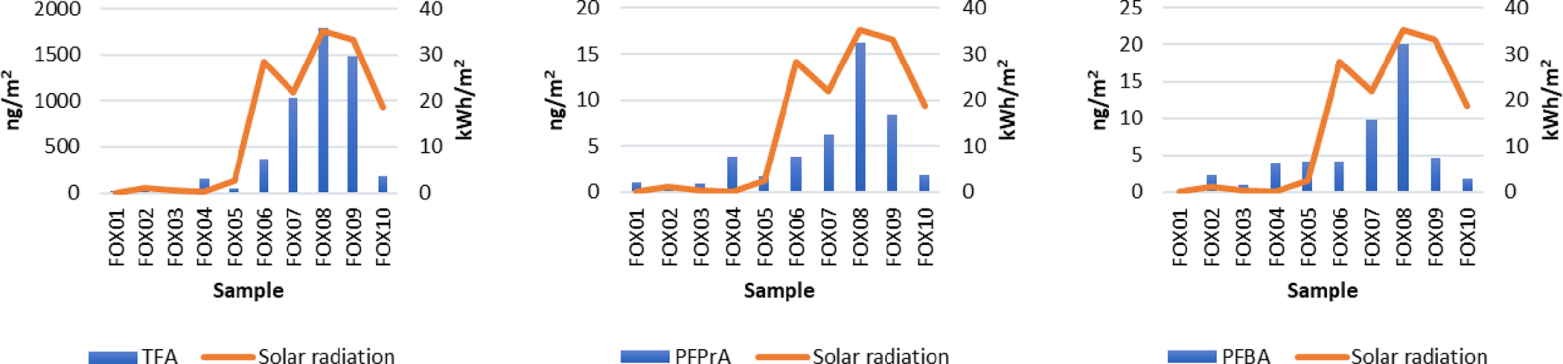
Flux (ng/m^2^) of TFA, PFPrA, and PFBA in surface snow samples collected at the Foxfonna ice cap from January 2019 (Fox01) to August 2019 (Fox10) and short-wave solar radiation (kWh/m^2^) along the backward trajectory.

Integrated values for the downward short-wave solar radiation along the 144 h (6 day) backward trajectory were found on average to be 39 times higher for samples Fox06–Fox10 (19.1–42.8 kWh/m^2^) compared to samples Fox01–Fox05 (0–2.5 kWh/m^2^). This is a result of the rapid shift from polar night to midnight sun at high latitudes during winter to summer. A positive correlation was found between the integrated downward short-wave solar radiation and the flux of TFA (*r* = 0.89, *P* < 0.01), PFPrA (*r* = 0.72, *P* < 0.05), and PFBA (*r* = 0.81, *P* < 0.01) (Figure 3 and Table S11). These findings suggest that the atmospheric transport of volatile precursors and the hydroxyl radical-driven atmospheric oxidation of these play a major role in the formation and subsequent deposition of C_2_–C_4_ PFCAs to the Arctic environment. The higher flux of PFAAs observed during summer compared to winter could also be influenced by factors such as temperature, humidity, and seasonal variations in emission rates. In addition, it is possible that solar radiation is correlated with air masses originating from source regions in Eurasia and that this could contribute to the correlations observed between solar radiation and increased flux of PFAAs. However, samples Fox07 and Fox09 all have backward air mass trajectories originating over the Arctic Ocean away from source regions and still have high TFA fluxes (Figure S1). This suggests that solar radiation is a more important factor than air mass source region.

### Discovery of TFMS at Foxfonna Ice Cap

TFMS was detected in all surface snow samples, including those collected at the reference locations, at fluxes in the range of 1.5–57 ng/m^2^. The detection of TFMS in surface snow at remote locations in the Arctic indicate that this compound may be globally distributed. To the best of our knowledge, this is the first study to report concentrations of TFMS not only in surface snow but also at remote locations. No seasonal variation was observed, and the concentrations were not related to solar radiation or notably higher at any sampling location. There are limited data available on the formation of C_1_–C_4_ PFSAs from volatile precursors. Formation of PFBS from the degradation of *N*-methyl perfluorobutane sulfonamidoethanol^25^ has been illustrated. However, the findings in the present study suggest that atmospheric sources other than atmospheric degradation of precursor compounds are involved in the long-range atmospheric transport of C_1_–C_4_ PFSAs to remote locations. To examine the influence of long-range oceanic transport on TFMS deposition at the Foxfonna sampling site, the flux of TFMS in surface snow samples from the Foxfonna ice cap was compared to the flux of sodium ions, which is a tracer for marine aerosols. No correlation was observed between sodium ions and TFMS (Table S11).

The PFAA flux in the current study is based on measured PFAA concentrations in a relatively low amount of snow events. Some studies have estimated that TFA is removed by dry deposition at a rate approximately 20% of the wet deposition rate, and hence, wet deposition is the dominating process for the removal of PFAAs from the atmosphere.^29,48^ The sampling was conducted as soon as possible after a certain precipitation event; in some cases, sampling was not possible until up to 7 days at Foxfonna. Transformation of precursor compounds in surface snow resulting in the formation of PFCAs has previously been suggested to explain higher concentrations of PFCAs in aged snow compared to fresh snow.^30^ This is likely to occur in presence of solar radiation and has not been accounted for in the present work. The current solar radiation assessment (using HYSPLIT) only accounts for the degradations that are occurring in the atmosphere rather than in the deposited snow. Other postdepositional effects are also not accounted such as for the revolatilization of PFAAs back into the atmosphere.^21^

C_1_–C_4_ PFAAs were frequently detected in snow samples, even in those collected at remote, high-altitude sites. The results suggest that hydroxyl radical-driven atmospheric oxidation plays a major role in the global distribution of C_2_–C_4_ PFCAs and the consequential deposition in Arctic environments. The discovery of TFMS in surface snow at remote locations indicates that TFMS could be globally distributed. However, the transport mechanism to the Arctic environment is still unknown. Despite the absence of correlation between C_1_–C_4_ PFSAs and solar radiation, the formation from degradation of volatile precursors cannot be ruled out, even if this source might be of minor importance. Atmospheric transport of TFMS to the Arctic could also be a particle-bound process. Further research is needed to investigate the formation of TFMS by atmospheric degradation of volatile precursors as well as the transport mechanism to remote locations.

## Supporting Information Available

List of target analytes, abbreviations, MRM transitions, and internal standards used for quantification, limits of detection, recoveries and repeatability, and Spearman rank correlations (PDF)
