# Supplementary material for: Levels and Seasonal Trends of C1–C4 Perfluoroalkyl Acids and the Discovery of Trifluoromethane Sulfonic Acid in Surface Snow in the Arctic
Source: Environ Sci Technol. 2021 Nov 15;55(23):15853–61. doi: 10.1021/acs.est.1c04776 (PMC8655978; doi:10.1021/acs.est.1c04776)
Supplement: Supplementary file 1 — es1c04776_si_001.pdf [file es1c04776_si_001.pdf]

Levels and seasonal trends of $C_1 – C_4$ perfluoroalkyl acids and the discovery of trifluoromethane sulfonic acid in surface snow in the Arctic

Maria K. Björnsdotter,[a]* William F. Hartz,[b,c,] Roland Kallenborn,[d,e] Ingrid Ericson Jogsten[a] Jack D. Humby,[f] Anna Kärrman,[a] Leo W. Y. Yeung,[a]

[a]Man-Technology-Environment Research Centre (MTM), Örebro University, SE-701 82 Örebro, Sweden

[b]Department of Earth Sciences, University of Oxford, South Parks Road, Oxford, OX1 3AN, United Kingdom

[c]Department of Arctic Geology, University Centre in Svalbard (UNIS), NO-9171, Longyearbyen, Svalbard, Norway

[d]Faculty of Chemistry, Biotechnology and Food Sciences (KBM), Norwegian University of Life Sciences (NMBU), NO-1432 Ås, Norway

[e]Department of Arctic Technology, University Centre in Svalbard (UNIS), NO-9171, Longyearbyen, Svalbard, Norway

[f]Ice Dynamics and Paleoclimate, British Antarctic Survey, High Cross, Cambridge, CB3 0ET, United Kingdom

*Corresponding author

E-mail: maria.bjornsdotter@oru.se

Number of tables: 11, number of figures: 1, number of pages: 11.

22 **Table of Content**

Table S1. List of target analytes, MRM transitions and internal standards used for quantification. S4

Table S2. Limits of quantification, extraction efficiency and repeatability, and instrument precision. S5

Table S3. Concentrations of C_1 – C_4 PFAAs (ng/L) and Sodium (Na^+) ions (ppb) in surface snow collected on the summit of Foxfonna ice cap (FOX), and the short-wave solar radiation (kWh/m^2), during January to August 2019. S6

Table S4. Concentrations of C_1 – C_4 PFAAs (ng/L) in surface snow collected up the hill from the Kjell Henriksen Observatory (KHO) during January to June 2019. S6

Table S5. Concentrations of C_1 – C_4 PFAAs (ng/L) in surface snow collected in Longyearbyen, outside the University Centre in Svalbard (UNIS) during January to May 2019. S7

Table S6. Concentrations of C_1 – C_4 PFAAs (ng/L) in surface snow collected at four high elevation sites on glaciers around Svalbard Spitsbergen during February to April 2019. S7

Table S7. Flux of C_1 – C_4 PFAAs (ng/m^2) and Sodium (Na^+) ions ($\mu g/m^2$) in surface snow collected on the summit of Foxfonna ice cap (FOX), and the short-wave solar radiation (kWh/m^2), during January to August 2019. S8

Table S8. Flux of C_1 – C_4 PFAAs (ng/m^2) in surface snow collected up the hill from the Kjell Henriksen Observatory (KHO) during January to June 2019. S8

Table S9. Flux of C_1 – C_4 PFAAs (ng/m^2) in surface snow collected in Longyearbyen, outside the University Centre in Svalbard (UNIS) during January to May 2019. S9

Table S10. Flux of C_1 – C_4 PFAAs (ng/m^2) in surface snow collected at four high elevation sites on glaciers around Svalbard Spitsbergen during February to April 2019. S9

Table S11. Spearman rank correlations. S10

Ion Chromatography for quantification of Na^+ concentrations S10

Figure S1. HYSPLIT backwards air mass trajectories for Fox01 – Fox10 and the hourly downward solar radiation along the trajectory (W/m^2). S11

Table S1. List of target analytes, IUPAC names, CAS numbers, abbreviations, MRM transitions and internal standards used for quantification.

Target analyte	IUPAC name	CAS	Abbreviation	1st MRM transition	2nd MRM transition	Internal standard
Trifluoroacetic acid	2,2,2-trifluoroethanoic acid	76-05-1	TFA	112.90 > 68.96	-	^{13}C-M2TFA
Perfluoropropanoic acid	2,2,3,3,3-pentafluoropropanoic acid	422-64-0	PFPrA	162.97 > 118.90	-	^{13}C-M4PFBA
Perfluorobutanoic acid	2,2,3,3,4,4,4-heptafluorobutanoic acid	375-22-4	PFBA	212.97 > 169.00	-	^{13}C-M4PFBA
Trifluoromethane sulfonic acid	1,1,1-trifluoromethane-1-sulfonic acid	1493-13-6	TFMS	149.12 > 79.91	149.12 > 98.95	^{13}C-M3PFBS
Perfluoroethane sulfonic acid	1,1,2,2,2-pentafluoroethane-1-sulfonic acid	354-88-1	PFEtS	198.80 > 79.80	198.80 > 98.90	^{13}C-M3PFBS
Perfluoropropane sulfonic acid	1,1,2,2,3,3,3-heptafluoropropane-1-sulfonic acid	423-41-6	PFPrS	248.9 > 79.90	248.9 > 98.90	^{13}C-M3PFBS
Perfluorobutane sulfonic acid	1,1,2,2,3,3,4,4,4-nonafluorobutane-1-sulfonic acid	375-73-5	PFBS	298.90 > 79.96	298.90 > 98.90	^{13}C-M3PFBS

Table S2. Instrument limits of quantification (LOQ) (ng/L), method limits of quantification (MQL) (ng/L). Extraction efficiency (%) and repeatability (%) based on spiked blank samples and spiked test samples (*n*=3). Repeatability (%) of the analytical method based on repeated standard injections (*n*=10).

	LOQ (ng/L)	MQL (ng/L)	Extraction efficiency (%)	Extraction method repeatability (%)	Analytical method repeatability (%)
TFA	0.009	0.009	121 ± 5	2.7	4.1
PFPrA	0.009	0.009	67 ± 8	11	2.4
PFBA	0.009	0.058	71 ± 6	14	3.0
TFMS	0.009	0.009	80 ± 16	15	2.8
PFEtS	0.009	0.045	81 ± 9	2.2	0.33
PFPrS	0.009	0.009	83 ± 8	6.0	2.4
PFBS	0.002	0.013	80 ± 12	3.3	1.7

Table S3. Concentrations of $C_1 - C_4$ PFAAs (ng/L) and sodium (Na^+) ions (ppb) in surface snow collected on the summit of the Foxfonna ice cap (Fox), and the downward short-wave solar radiation (kWh/m^2), during January to August 2019.

	TFA	PFPrA	PFBA	TFMS	PFEtS	PFPrS	PFBS	Na^+ (ppb)	Solar radiation (kWh/m^2)
Fox01	7.4	0.31	<0.06	1.3	<0.05	<0.009	<0.01	660	0.0
Fox02	13	0.36	1.1	5.1	<0.05	<0.009	<0.01	600	1.3
Fox03	7.3	0.29	0.33	0.92	<0.05	<0.009	<0.01	480	0.0
Fox04	16	0.41	0.43	1.0	<0.05	<0.009	<0.01	700	0.37
Fox05	11	0.41	0.98	0.53	<0.05	<0.009	<0.01	190	2.5
Fox06	54	0.57	0.61	0.63	<0.05	<0.009	<0.01	17	27
Fox07	170	1.0	1.6	0.36	<0.05	<0.009	<0.01	240	33
Fox08	170	1.5	1.9	0.29	<0.05	<0.009	<0.01	430	43
Fox09	270	1.5	0.84	1.7	<0.05	<0.009	<0.01	650	41
Fox10	49	0.51	0.49	1.3	<0.05	<0.009	<0.01	460	19

Table S4. Concentrations of $C_1 - C_4$ PFAAs (ng/L) in surface snow collected up the hill from the Kjell Henriksen Observatory (KHO) during January to June 2019.

	TFA	PFPrA	PFBA	TFMS	PFEtS	PFPrS	PFBS
KHO01	5.6	0.21	0.57	1.0	0.10	<0.009	<0.01
KHO02	16	0.54	1.1	1.3	0.11	<0.009	<0.01
KHO03	7.7	0.32	0.10	3.0	<0.05	<0.009	<0.01
KHO04	12	0.29	0.35	1.9	<0.05	<0.009	<0.01
KHO05	17	0.46	0.34	0.97	<0.05	<0.009	<0.01
KHO06	80	0.55	0.62	0.50	<0.05	<0.009	<0.01
KHO07	100	1.0	0.56	0.15	<0.05	<0.009	<0.01
KHO08	93	0.96	0.90	0.22	<0.05	<0.009	<0.01
KHO09	73	0.89	1.0	0.22	<0.05	<0.009	<0.01

Table S5. Concentrations of $C_1 – C_4$ PFAAs (ng/L) in surface snow collected in Longyearbyen, outside the University Centre in Svalbard (UNIS) during January to May 2019.

	TFA	PFPrA	PFBA	TFMS	PFEtS	PFPrS	PFBS
UNIS01	18	0.66	3.4	2.6	0.24	<0.009	2.4
UNIS02	24	0.74	3.6	2.4	0.71	<0.009	0.1
UNIS03	16	0.46	10	2.1	0.35	<0.009	<0.01
UNIS04	70	0.86	2.8	1.3	0.22	<0.009	<0.01
UNIS05	27	1.2	2.7	1.2	0.29	<0.009	<0.01
UNIS06	110	0.75	2.0	1.0	0.24	<0.009	0.03
UNIS07	190	1.1	6.2	0.65	0.26	<0.009	0.07
UNIS08	50	0.72	8.8	2.9	2.8	<0.009	0.09

Table S6. Concentrations of $C_1 – C_4$ PFAAs (ng/L) in surface snow collected at four high elevation sites on glaciers around Spitsbergen during February to April 2019.

	TFA	PFPrA	PFBA	TFMS	PFEtS	PFPrS	PFBS
Lomonosovfonna01	18	0.34	0.37	0.37	<0.05	<0.009	<0.01
Lomonosovfonna02	30	0.53	0.57	0.89	<0.05	<0.009	<0.01
Drønbreen	13	0.40	0.58	1.1	<0.05	<0.009	<0.01
Grønfjordbreen	90	1.1	0.82	1.5	<0.05	<0.009	<0.01
Nordmannsfonna	62	0.75	0.63	1.5	<0.05	<0.009	<0.01

Table S7. Flux of $C_1 – C_4$ PFAAs (ng/m^2) and Sodium (Na^+) ions (µg/m^2) in surface snow collected on the summit of the Foxfonna ice cap (Fox), and the downward short-wave solar radiation (kWh/m^2), during January to August 2019.

	TFA	PFPrA	PFBA	TFMS	PFEtS	PFPrS	PFBS	Na^+ (µg/m^2)	Solar radiation (kWh/m^2)
Fox01	23	0.99	<0.19	4.0	<0.14	<0.03	<0.04	2100	0.0
Fox02	28	0.79	2.4	11	<0.10	<0.02	<0.03	1300	1.1
Fox03	22	0.87	0.99	2.8	<0.14	<0.03	<0.04	1400	0.41
Fox04	150	3.9	4.0	9.7	<0.42	<0.09	<0.12	6600	0.18
Fox05	46	1.7	4.2	2.3	<0.19	<0.04	<0.05	800	2.6
Fox06	360	3.8	4.1	4.2	<0.30	<0.06	<0.09	120	28
Fox07	1000	6.2	9.8	2.2	<0.28	<0.06	<0.08	1500	22
Fox08	1800	16	20	3.1	<0.48	<0.10	<0.14	4600	35
Fox09	1500	8.3	4.6	9.4	<0.25	<0.05	<0.07	3600	33
Fox10	180	1.8	1.8	4.9	<0.16	<0.03	<0.05	1700	19

Table S8. Flux of $C_1 – C_4$ PFAAs (ng/m^2) in surface snow collected up the hill from the Kjell Henriksen Observatory (KHO) during January to June 2019.

	TFA	PFPrA	PFBA	TFMS	PFEtS	PFPrS	PFBS
KHO01	82	3.0	8.3	15	1.5	<0.13	<0.19
KHO02	72	2.5	4.8	6.1	0.52	<0.04	<0.06
KHO03	41	1.7	0.51	16	<0.24	<0.05	<0.07
KHO04	123	2.9	3.5	19	<0.45	<0.09	<0.13
KHO05	89	2.4	1.8	5.1	<0.24	<0.05	<0.07
KHO06	690	4.7	5.3	4.3	<0.39	<0.08	<0.11
KHO07	1000	11	5.7	1.5	<0.46	<0.09	<0.13
KHO08	640	6.6	6.1	1.5	<0.31	<0.06	<0.09
KHO09	560	6.8	7.9	1.6	<0.34	<0.07	<0.10

Table S9. Flux of $C_1 – C_4$ PFAAs (ng/m^2) in surface snow collected in Longyearbyen, outside the University Centre in Svalbard (UNIS) during January to May 2019.

	TFA	PFPrA	PFBA	TFMS	PFEtS	PFPrS	PFBS
UNIS01	190	7.1	36	28	2.6	<0.10	26
UNIS02	220	6.7	32	22	6.4	<0.08	0.93
UNIS03	120	3.3	75	15	2.5	<0.07	<0.09
UNIS04	480	5.9	19	9.0	1.5	<0.06	<0.09
UNIS05	140	6.2	14	6.3	1.5	<0.05	<0.07
UNIS06	720	4.8	13	6.4	1.5	<0.06	0.17
UNIS07	1800	10	59	6.2	2.5	<0.09	0.65
UNIS08	980	14	170	57	55	<0.18	1.7

Table S10. Flux of $C_1 – C_4$ PFAAs (ng/m^2) in surface snow collected at four high elevation sites on glaciers around Spitsbergen during February to April 2019.

	TFA	PFPrA	PFBA	TFMS	PFEtS	PFPrS	PFBS
Lomonosovfonna01	160	3.0	3.3	3.3	<0.40	<0.08	<0.11
Lomonosovfonna02	330	5.9	6.3	9.9	<0.50	<0.10	<0.14
Drønbreen	66	2.0	3.0	5.8	<0.23	<0.05	<0.07
Grønfjordbreen	710	8.9	6.5	12	<0.35	<0.07	<0.10
Nordmannsfonna	210	2.6	2.1	5.1	<0.15	<0.03	<0.04

Table S11. Spearman rank correlations (r) between the flux of TFA, PFPrA, PFBA and TFMS (ng/m^2); Na^+ (µg/m^2) and solar radiation (kWh/m^2).

	TFA		PFPrA		PFBA		TFMS	
	r	p	r	p	r	p	r	p
PFPrA	**0.93**	**<0.01**						
PFBA	**0.85**	**<0.01**	**0.81**	**<0.01**				
TFMS	-0.03	0.93	-0.12	0.75	-0.27	0.45		
Solar radiation	**0.89**	**<0.01**	**0.72**	**<0.05**	**0.81**	**<0.01**	-0.14	0.70
Na^+	0.33	0.35	0.55	0.10	0.14	0.70	0.26	0.47

Ion Chromatography for quantification of Na^+ concentrations

Na^+ concentrations were measured using a Dionex ICS-4000 Integrion system. A Dionex AS-AP autosampler was used to supply sample to the instrument. Calibration of IC data was achieved using a range of calibration standards. All calibration standards were prepared from purchased Sigma Aldrich (1000 ppm) standards by a series of gravimetric dilutions.

The columns used were a Dionex Ionpac CG16-4µm (2 x 50 mm) guard column and CS16 (4 µm, 2 x 250 mm) analytical column. Samples were loaded to a 250 µL sample loop. A 32 – 42 mM methane sulfonic acid (MSA) multistep eluent gradient was used for effective separation of the analytes. The eluent was produced using a Dionex eluent generator cartridge (EGC 500 KOH) and was pumped through the columns at a flow rate of 0.20 mL min^{-1} at a pressure of 3300 psi for a 25 min chromatogram. The eluent ions were removed from the column effluent using a cation electrolytically regenerated suppressor (Dionex CERS 500), before conductivity detection of the effluent.

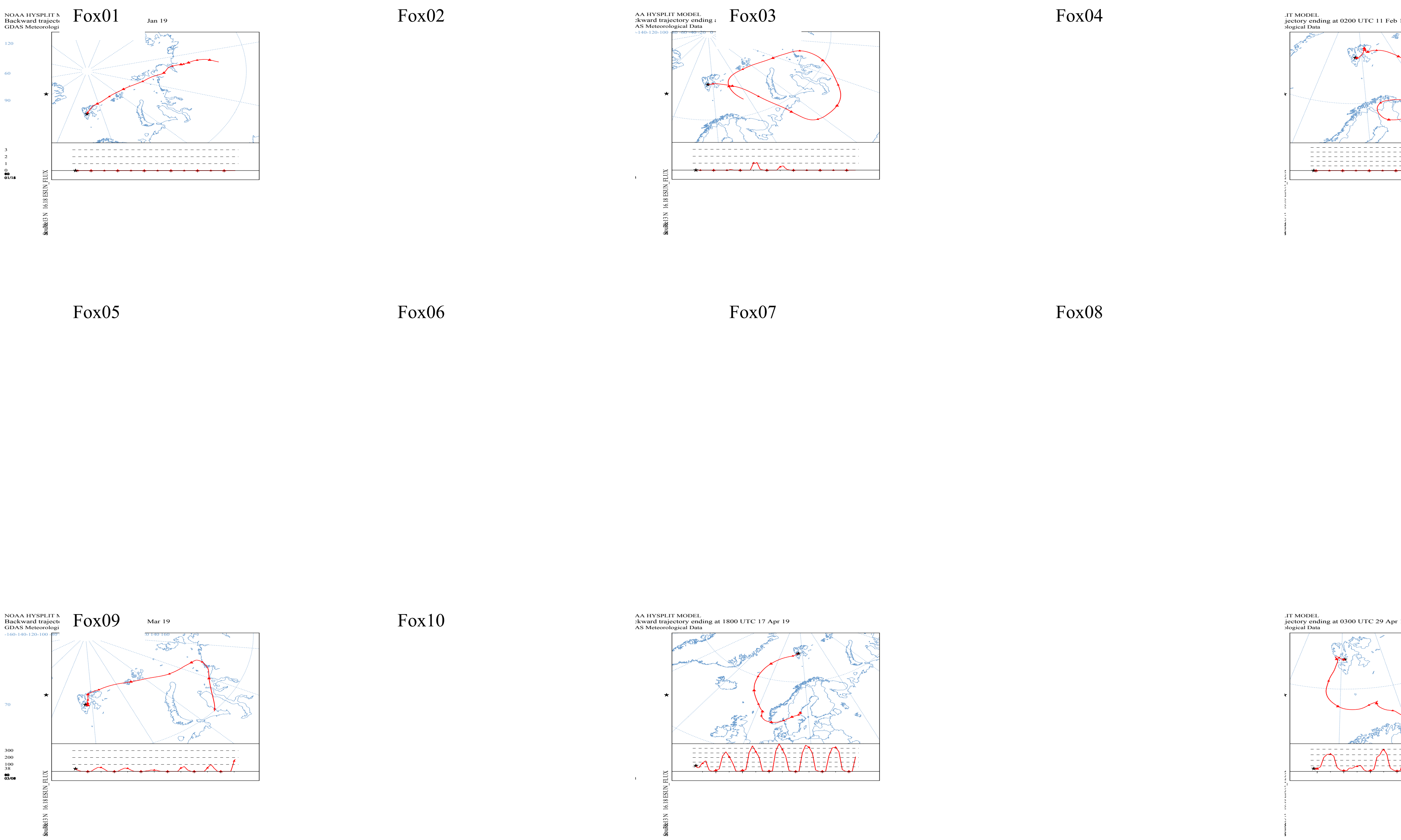

Figure S1. HYSPLIT backwards air mass trajectories for Fox01 – Fox10 and the hourly downward solar radiation along the trajectory (W/m^2).
